# Exercise Training Duration and Intensity Are Associated With Thicker Carotid Intima-Media Thickness but Improved Arterial Elasticity in Active Children and Adolescents

**DOI:** 10.3389/fcvm.2021.618294

**Published:** 2021-07-08

**Authors:** Lisa Baumgartner, Heidi Weberruß, Tobias Engl, Thorsten Schulz, Renate Oberhoffer-Fritz

**Affiliations:** Institute of Preventive Pediatrics, TUM Department of Sport and Health Science, Technical University of Munich, Munich, Germany

**Keywords:** arterial elasticity, arterial stiffness, exercise load, carotid intima - media thickness, youth

## Abstract

Even though exercise generally has a positive effect on health, intensive exercise can have adverse effects on the vascular system of adults. This study aimed to investigate the association between training duration and intensity and vascular structure and function in 427 physically active children and adolescents (14.0 ± 1.94 years). In this study, we examined carotid intima-media thickness (cIMT), carotid diameter, and cIMT:diameter-ratio as parameters of carotid arterial structure and arterial compliance (AC), stiffness index β (β), elastic modulus (Ep), and carotid pulse wave velocity (PWVβ) as parameters of carotid arterial function with high-resolution ultrasound. We collected central systolic blood pressure (cSBP) and aortic pulse wave velocity (aPWV) as parameters of central arterial stiffness with an oscillometric device. We used the MoMo Physical Activity Questionnaire to record training duration and intensity. Training duration (*p* = 0.022) and intensity (*p* = 0.024) were associated with higher cIMT. Further, training duration was associated with lower central arterial stiffness (cSBP: *p* = 0.001; aPWV: *p* = 0.033) and improved AC (*p* < 0.001). Higher training intensity was related to improved AC (*p* < 0.001) and larger carotid diameter (*p* = 0.040). Boys presented thicker cIMT (*p* = 0.010), improved AC (*p* = 0.006), and lower central arterial stiffness (cSBP: *p* < 0.001; aPWV: *p* = 0.016) associated with higher training duration. Girls presented improved AC (*p* = 0.023) and lower Ep (*p* = 0.038) but higher β (*p* = 0.036) associated with higher training duration. Only boys demonstrated thicker cIMT (*p* = 0.016) and improved AC (*p* = 0.002) associated with higher training intensity. A quintile analyses of the training duration revealed thicker cIMT of children and adolescents in Q1 and Q5 than that in Q4 and Q5. Besides, Q1 showed lower cSBP compared to Q4 and Q5. Regarding training intensity, Q5 had thicker cIMT than Q2 and Q3. Although a higher training load is associated with thicker cIMT, the common carotid artery is also more elastic. This suggests that a higher training load leads to a functional adaptation of the carotid artery in youth.

## Introduction

In adults, greater exposure to cardiovascular risk factors such as obesity is related to thicker arterial walls, and exercise training is associated with a thinner arterial wall (1). Similarly, exercise training is associated with improved vascular function and the presence of cardiovascular risk factors with reduced vascular function (2). The term “athlete's artery” summarizes the vascular adaptations to exercise, analogous to cardiac adaptations to exercise and the term “athlete's heart.” The “athlete's artery” is characterized by lower wall thickness, higher lumen or diameter, and lower arterial stiffness (3). Arterial structure and function measurements are divided into central (aorta) and peripheral (e.g., carotid or brachial artery) measurements.

Even though exercise generally has a positive effect on health, high volume exercise can have adverse effects on the vascular system. Müller et al. (4) found a significant increase in carotid intima-media thickness (cIMT) in male marathon runners within 4 years, which was contrary to the postulated reduced wall thickness. Another study showed that higher central systolic blood pressure (cSBP) and aortic pulse wave velocity (aPWV) were observed in predominantly male marathon runners (5). Also, strength-trained men showed higher arterial stiffness that may depict early subclinical vascular dysfunction (6–8).

Also, training frequency and training volume have a significant effect on the extent of cardiovascular adaptation (6, 9, 10). Aengevaeren et al. (10) investigated the extent of coronary artery calcification (CAC), plaque characteristics, and exercise volumes [metabolic equivalent of task (MET)] in men who participated in competitive and recreational sports. They observed an odds ratio (OR) of 3.3 in the prevalence of atherosclerotic plaques, with a training volume of >2,000 MET-min/week, and an OR of 3.2 with a CAC score of > 0 compared with persons with a volume of <1,000 MET-min/week. Recently, studies have also been performed in pediatric populations for the first time. A study observed better carotid elasticity but higher cIMT and cIMT÷carotid diameter-ratio (cIDR) and higher central arterial stiffness in young athletes (11). Another study found lower cIMT in male adolescent wrestlers than in controls, but there were no differences in cIMT and vascular function observed in young male athletes participating in dynamic or strength sports (12, 13).

There is no evidence of the impact of training behavior on vascular health in the overall population of healthy active children and adolescents although overall physical activity is considered to have a positive effect on vascular properties in childhood and adolescence (14). Besides, the studies conducted so far have mainly involved male subjects. Therefore, to what extent vascular properties differ between young active girls and boys and whether exercise in sports clubs has a different effect on vascular properties between sexes is not clear. Furthermore, the relationship between different training frequencies and intensities on the vascular system in youth has not been studied sufficiently.

Thus, this study aimed to investigate the association between training duration and intensity and the vascular structure and function in physically active children and adolescents, overall and separated for boys and girls. We hypothesized that there is an association between higher training duration and intensity and better vascular properties in both sexes. We also examined the differences in vascular properties between quintiles of training duration and intensity to study whether vascular properties follow a curved pattern associated with higher training load.

## Methods

### Study Design and Study Participants

In our pediatric department, a total of 734 children and adolescents, regularly exercising in sports club activities, were enrolled in a pre-participation screening. The recruitment started in November 2017 and lasted until September 2020. Of these 734 subjects, 108 were examined twice, 32 three times and 3 four times, as these subjects received annual pre-participation screening. In this case, only the first examination was considered and the 181 multiple measurement were not included. In 88 subjects, the vascular measurements could not be performed and we excluded 38 subjects because of their age (<8.00 or >17.25 years). Thus, the analyzed dataset was comprised of 427 healthy participants (25.0% females) aged between 8 and 17 years.

The data collection was part of the Munich Cardiovascular Adaptation in Young Athletes study (MuCAYA study, data from September 2018 to September 2020) and the upstream pilot study (data from November 2017 to September 2018) (15). Both studies were approved by the local ethics committee and conducted in accordance with the Declaration of Helsinki (projects numbers 301/18 S and 131/19 S-SR). All children and/or their legal guardians provided written informed consent before the examinations.

### Assessment of Body Size and Body Surface Area

We measured the body height and body mass of the participants without shoes, wearing light sports clothing in an upright position, with straight shoulders, and looking ahead. The body mass was determined precisely at 0.1 kg and body size at 0.1 cm (seca 799, seca GmbH & Co. KG, Hamburg, Germany). The body mass index (BMI) was calculated by dividing body mass (kg) by body height squared (m^2^). Based on the German reference data, standard deviation score (SDS) of BMI was calculated (16). According to Dubois and Dubois (17), the body surface area (BSA) was calculated.

### Assessment of Blood Pressure and Central Arterial Stiffness

Using the oscillometric device Mobil-O-Graph (IEM, Stolberg, Germany), brachial systolic blood pressure (bSBP), diastolic blood pressure (bDBP), cSBP, and aPWV were measured automatically on the left arm in a supine position after 10 min of rest. Depending on the circumference of the upper arm, the cuff size was selected. cSBP of the Mobil-O-Graph device has been validated in comparison to the SphycmoCor device and radial tonometry (18, 19) and aPWV in comparison to intracatheter measurements (20). cSBP and aPWV were computed using the ARCSolver algorithm (Austrian Institute of Technology, Vienna, Austria).

### Assessment of Carotid Structure and Function

We used semi-automated ultrasound with a high frequency linear array probe of 5–13 MHz (Aloka Prosound α7, Hitachi Medical Systems GmbH, Wiesbaden, Germany) to measure carotid structure and function in a supine position after 10 min of rest. Carotid arterial properties, were taken from the left and right common carotid artery (CCA), 1 cm proximal to the carotid bulb using B- and M-Mode ultrasound. On each side, cIMT was measured during diastole at two angles (right, 120° and 150°; left, 240° and 210°) at the end of diastole on the CCA far wall (15). Furthermore, carotid diameter and function were examined using the eTracking method twice on each side (right, 150°; left, 210°). The carotid diameter was measured in systole (D_max_) and diastole (D_min_) but only D_min_ was used for further analysis and calculation of cIDR, corresponding to cIMT measured at diastole, and is denoted as carotid diameter in this document. Parameters of carotid function, including arterial compliance (AC), elastic modulus (Ep), stiffness index β (β), and local PWV at the CCA (PWVβ), were calculated using the following formula (15, 21–24):

(1) $$\begin{array}{l} AC\;=\;\pi \;\frac{\left(D_{\overset{2}{\max}}-\;D_{\overset{2}{\min}}\right)}{4\;\left(bSBP_{\max}-\;bDBP_{\min}\right)} \end{array}$$

(2) $$\begin{array}{l} Ep\;=\;\frac{bSBP_{\max}-\;bDBP_{\min}}{\frac{D_{\max}-\;D_{\min}}{D_{\min}}} \end{array}$$

(3) $$\begin{array}{l} \beta \;=\;\frac{\ln\frac{bSBP_{\max}}{bDBP_{\min}}}{\frac{D_{\max}-\;D_{\min}}{D_{\min}}} \end{array}$$

(4) $$\begin{array}{l} PWV\beta \;=\;\sqrt{\frac{\beta *bDBP_{\min}}{2\rho }} \end{array}$$

All parameters were calculated as average values of four measurements. All measurements were conducted by two investigators. The analysis of the inter-observer variability revealed a small to acceptable deviation (11).

### Assessment of Training Duration and Intensity

All children and adolescents completed the MoMo Physical Activity Questionnaire (MoMo-PAQ) for pupils (25, 26). The questionnaire records self-reported physical activity in different settings (school, everyday activities, sports clubs, leisure-time outside of sports clubs) regarding the amount (min/day) and intensity (low, moderate, intense), which allows the calculation of sports-specific MET values (27). Physical activity at school, everyday activities, and leisure time physical activity outside of sports clubs were not considered, because this study focuses only on the training behavior in sports clubs but not on the overall activity in all settings.

The estimated intraclass correlation for reliability of sports club activities was 0.64 (*p* < 0.01) in overall, 0.49 (*p* < 0.01) for boys, 0.78 (*p* < 0.01) for girls, 0.45 (*p* < 0.01) for children, and 0.90 (*p* < 0.01) for adolescents (28). Validity was assessed in comparison with the Actigraph GT1M activity monitor (*r* = 0.35, *p* < 0.01) and the Previous Day Physical Activity Recall self-report instrument (*r* = 0.55, *p* < 0.01) (28).

Our subjects reported the weekly amount of physical activity spent in the type of sport (without outward and return, changing clothes, and showering) and the number of months in which the weekly amount of physical activity was performed. We calculated the weekly minutes spent in sports clubs and thus training duration using the following formula:

(5) $$\begin{array}{l} training\;duration\;=\;minutes\;per\;week\;*\frac{number\;of\;months}{12} \end{array}$$

Depending on the reported intensity level, an individual MET score was assigned to every recorded type of sport (27, 29, 30). MET-min/week spent in sports clubs was calculated and then divided by 60 to obtain the MET-hours-index:

(6) $$\begin{array}{l} training\;intensity\;=\;\frac{training\;duration\;*MET}{60} \end{array}$$

To assess training history, the participants were asked how many years they had been performing their major type of sport.

### Statistical Analysis

We used SPSS version 25.0 (SPSS Inc., Chicago, IL, USA) for all statistical analyses. The level of significance was set at *p* < 0.05. Continuous variables are presented as mean ± standard deviation and categorical variables as n (%). Sex differences were analyzed using an independent *t*-test. The association between training duration and intensity and vascular parameters was assessed using multiple linear regression analyses. All regression models were adjusted for age, sex, BSA, and training years (31–33). Regression models with parameters of carotid arterial structure were adjusted further for bSBP (31). All regression models were repeated separately for boys and girls. The differences in parameters of vascular structure and function between quintiles of training duration and intensity were examined using analysis of covariance (ANCOVA) with Bonferroni *post hoc* tests. Based on the distribution of training duration and intensity within the dataset, the classification in quintiles was conducted. Regarding the quintiles of training duration, Q1 referred to a training duration of up to 330.0 min/week, Q2 from 330.1 to 412.5 min/week, Q3 from 412.6 to 481.5 min/week, Q4 from 481.6 to 626.0 min/week, and Q5 above 626.0 min/week. Regarding the quintiles of training intensity, Q1 referred to a training intensity of up to 49.5 MET-hours-index, Q2 from 49.6 to 64.2 MET-hours-index, Q3 from 64.3 to 80.0 MET-hours-index, Q4 from 80.1 to 99.0 MET-hours-index, and Q5 above 99.0 MET-hours-index. The ANCOVAs were adjusted for the same variables as in the regression models.

## Results

The study collective had a mean age of 14.0 ± 1.94 years and spent 491.9 ± 224.7 min/week in sports club activities (Table 1). Children and adolescents participated in 36 different types of sports. Soccer (36.8%), volleyball (9.4%), hockey (8.7%), wrestling (6.8%), and cross-country skiing (5.6%) were the main types of sports. Active boys had significantly higher body height, body mass, BSA, bSBP, cIMT, carotid diameter, cIDR, AC, cSBP, and aPWV than girls (Table 1). BMI SDS was 0.001 ± 0.802 in boys and 0.078 ± 0.777 in girls. Of 427 subjects, 26 (6.1%) had isolated bSBP >95th percentile, one (0.2%) had isolated bDBP >95th percentile, and five (1.2%) subjects presented combined bSBP and bDBP >95th percentile (34).

**Table 1:** Study characteristics.

	**Total**		**Boys**		**Girls**		***p***
	***n***	**M ± SD**	***n***	**M ± SD**	***n***	**M ± SD**	
Age (y)	427	14.0 ± 1.94	320	14.0 ± 1.94	107	13.9 ± 1.96	0.458
Body mass (kg)	427	55.4 ± 14.6	320	56.2 ± 15.4	107	53.0 ± 11.9	**0.024**
Body height (cm)	427	166.2 ± 14.5	320	167.6 ± 15.1	107	161.9 ± 11.8	** <0.001**
BMI (kg/m^2^)	427	19.7 ± 2.72	320	19.6 ± 2.72	107	19.9 ± 2.74	0.272
BMI SDS	427	0.020 ± 0.796	320	0.001 ± 0.802	107	0.078 ± 0.777	0.384
BSA (m^2^)	427	1.59 ± 0.28	320	1.61 ± 0.29	107	1.54 ± 0.23	**0.008**
bSBP (mm Hg)	427	115.0 ± 10.2	320	115.8 ± 10.9	107	112.7 ± 7.74	**0.002**
bDBP (mm Hg)	427	65.2 ± 6.26	320	65.2 ± 6.35	107	65.2 ± 6.00	0.978
Training duration (min/week)	427	491.9 ± 224.7	320	489.9 ± 212.3	107	495.7 ± 259.3	0.856
Training intensity (MET-hours-index)	424	76.4 ± 36.1	318	76.9 ± 31.8	106	74.7 ± 46.9	0.640
Training years (y)	395	3.43 ± 2.53	296	3.54 ± 2.64	99	3.13 ± 2.24	0.152
cIMT (mm)	427	0.47 ± 0.04	320	0.48 ± 0.04	107	0.46 ± 0.04	** <0.001**
Carotid diameter (mm)	422	5.63 ± 0.46	316	5.66 ± 0.47	106	5.52 ± 0.42	**0.006**
cIDR	422	0.085 ± 0.009	316	0.085 ± 0.009	106	0.083 ± 0.009	**0.039**
AC (mm^2^/kPa)	422	1.52 ± 0.33	316	1.55 ± 0.33	106	1.45 ± 0.31	**0.008**
β	422	3.20 ± 0.74	316	3.18 ± 0.73	106	3.28 ± 0.81	0.273
Ep (kPa)	422	37.5 ± 9.11	316	37.4 ± 8.86	106	38.2 ± 10.0	0.423
PWVβ (m/s)	422	3.61 ± 0.39	316	3.60 ± 0.38	106	3.65 ± 0.44	0.259
cSBP (mm Hg)	427	105.1 ± 10.8	320	105.9 ± 11.4	107	102.5 ± 8.40	**0.001**
aPWV (m/s)	427	4.89 ± 0.45	320	4.93 ± 0.48	107	4.74 ± 0.32	** <0.001**

*BMI, Body mass index; SDS, standard deviation score; BSA, body surface area; bSBP, brachial systolic blood pressure; bDBP, brachial diastolic blood pressure; MET, metabolic equivalent; cIMT, carotid intima-media thickness; cIDR, carotid intima-media thickness:carotid diameter-ratio; AC, arterial compliance; β, beta stiffness index; Ep, elastic modulus; PWVβ, carotid pulse wave velocity; cSBP, central systolic blood pressure; aPWV, aortic pulse wave velocity. Significant results are marked in bold*.

Multiple linear regressions with training duration revealed a significant positive association with cIMT (std. β = 0.113, *p* = 0.022), but not with carotid diameter or cIDR (Table 2). Higher training duration was also related to higher AC (std. β = 0.184, *p* = < 0.001), but not to parameters of carotid arterial stiffness. A higher number of minutes spent in sports club activities was associated with lower cSBP (std. β = −0.139, *p* = 0.001) and aPWV (std. β = −0.085, *p* = 0.033).

**Table 2:** Association between training duration (min/week) and vascular properties in active children and adolescents.

	***n***	**β**	**Std. β**	***p***	***R^**2**^***
cIMT^*^ (mm)	395	0.00002	0.113	**0.022**	0.183
Carotid diameter^*^ (mm)	392	0.0002	0.081	0.111	0.122
cIDR^*^	392	0.000001	0.030	0.573	0.021
AC^+^ (mm^2^/kPa)	392	0.0003	0.184	** <0.001**	0.116
β^+^	392	−0.0002	−0.046	0.366	0.084
Ep^+^ (kPa)	392	−0.004	−0.094	0.061	0.121
PWVβ^+^ (m/s)	392	−0.0002	−0.092	0.068	0.112
cSBP^+^ (mm Hg)	395	−0.007	−0.139	**0.001**	0.387
aPWV^+^ (m/s)	395	−0.0002	−0.085	**0.033**	0.434

*cIMT, carotid intima-media thickness; cIDR, carotid intima-media thickness:carotid diameter-ratio; AC, arterial compliance; β, beta stiffness index; Ep, elastic modulus; PWVβ, carotid pulse wave velocity; cSBP, central systolic blood pressure; aPWV, aortic pulse wave velocity. Significant results are marked in bold*.

* *Adjusted for age, sex, body surface area, training years, and bSBP*.

+ *Adjusted for age, sex, body surface area, and training years*.

Multiple linear regressions with training intensity revealed a significant positive association with cIMT (std. β = 0.111, *p* = 0.024) and carotid diameter (std. β = 0.105, *p* = 0.040), but not with cIDR (Table 3). Higher training intensity was also related to higher AC (std. β = 0.179, *p* < 0.001), but not to carotid arterial stiffness. Neither of central arterial stiffness parameters (cSBP and aPWV) was associated with training intensity.

**Table 3:** Association between training intensity (MET-hours-index) and vascular properties in active children and adolescents.

	***n***	**β**	**Std. β**	***p***	***R^**2**^***
cIMT^*^ (mm)	393	0.0001	0.111	**0.024**	0.183
Carotid diameter^*^ (mm)	390	0.001	0.105	**0.040**	0.127
cIDR^*^	390	0.000003	0.013	0.805	0.019
AC^+^ (mm^2^/kPa)	390	0.002	0.179	** <0.001**	0.115
β^+^	390	−0.001	−0.033	0.528	0.083
Ep^+^ (kPa)	390	−0.016	−0.062	0.224	0.116
PWVβ^+^ (m/s)	390	−0.001	−0.053	0.298	0.107
cSBP^+^ (mm Hg)	393	−0.018	−0.060	0.162	0.372
aPWV^+^ (m/s)	393	−0.0004	−0.028	0.493	0.427

*cIMT, carotid intima-media thickness; cIDR, carotid intima-media thickness:carotid diameter-ratio; AC, arterial compliance; β, beta stiffness index; Ep, elastic modulus; PWVβ, carotid pulse wave velocity; cSBP, central systolic blood pressure; aPWV, aortic pulse wave velocity. Significant results are marked in bold*.

* *Adjusted for age, sex, body surface area, training years, and bSBP*.

+ *Adjusted for age, sex, body surface area, and training years*.

Multiple linear regression models were performed separated for boys and girls for identifying sex-specific differences in the association between vascular parameters and training duration and intensity (Table 4). A positive association between training duration and cIMT was found in boys (std. β = 0.151, *p* = 0.010), but not in girls. Neither boys nor girls showed a correlation between carotid diameter and cIDR and training duration, but boys (std. β = 0.161, *p* = 0.006) and girls (std. β = 0.250, *p* = 0.023) revealed a positive correlation between AC and training duration. Regarding the parameters of carotid arterial stiffness, a negative correlation between β (std. β = −0.226, *p* = 0.036) and Ep (std. β = −0.222, *p* = 0.038) and training duration was found only in girls. On the other hand, a negative association between cSBP (std. β = −0.179, *p* < 0.001) and aPWV (std. β = −0.130, *p* = 0.005) and training duration was found only in boys. Concerning training intensity, positive correlations with cIMT (std. β = 0.139, *p* = 0.016) and AC (std. β = 0.177, *p* = 0.002) were also found only in boys.

**Table 4:** Sex-specific associations between training duration (min/week) and training intensity (MET-hours-index) and vascular properties in active children and adolescents.

		**Boys**					**Girls**				
		***n***	**β**	**Std. β**	***p***	***R^**2**^***	***n***	**β**	**Std. β**	***p***	***R^**2**^***
Training duration (min/week)	cIMT^*^ (mm)	296	0.00003	0.151	**0.010**	0.151	99	0.000002	0.014	0.899	0.064
	Carotid diameter^*^ (mm)	294	0.0003	0.109	0.070	0.105	98	−0.00005	−0.028	0.793	0.179
	cIDR^*^	294	0.000002	0.045	0.475	0.019	98	0.0000007	0.020	0.859	0.078
	AC^+^ (mm^2^/kPa)	294	0.0003	0.161	**0.006**	0.122	98	0.0003	0.250	**0.023**	0.102
	β^+^	294	0.00007	0.021	0.729	0.077	98	−0.001	−0.226	**0.036**	0.135
	Ep^+^ (kPa)	294	−0.002	−0.055	0.341	0.121	98	−0.009	−0.222	**0.038**	0.152
	PWVβ^+^ (m/s)	294	−0.0001	−0.059	0.312	0.099	98	−0.0002	−0.204	0.054	0.164
	cSBP^+^ (mm Hg)	296	−0.010	−0.179	** <0.001**	0.433	99	−0.0004	−0.011	0.916	0.168
	aPWV^+^ (m/s)	296	−0.0003	−0.130	**0.005**	0.455	99	0.0001	0.081	0.407	0.270
Training intensity (MET-hours-index)	cIMT^*^ (mm)	295	0.0002	0.139	**0.016**	0.150	98	0.00006	0.085	0.460	0.070
	Carotid diameter^*^ (mm)	293	0.001	0.089	0.132	0.102	97	0.001	0.089	0.409	0.186
	cIDR^*^	293	0.00002	0.053	0.369	0.020	97	−0.000003	−0.018	0.878	0.078
	AC^+^ (mm^2^/kPa)	293	0.002	0.177	**0.002**	0.128	97	0.001	0.158	0.169	0.070
	β^+^	293	−0.0003	−0.013	0.823	0.077	97	−0.002	−0.089	0.432	0.099
	Ep^+^ (kPa)	293	−0.018	−0.064	0.262	0.122	97	−0.017	−0.078	0.482	0.116
	PWVβ^+^ (m/s)	293	−0.001	−0.064	0.269	0.100	97	−0.001	−0.058	0.598	0.132
	cSBP^+^ (mm Hg)	295	−0.029	−0.082	0.082	0.409	98	0.008	0.043	0.686	0.170
	aPWV^+^ (m/s)	295	−0.001	−0.057	0.214	0.441	98	0.001	0.117	0.244	0.276

*cIMT, carotid intima-media thickness; cIDR, carotid intima-media thickness:carotid diameter-ratio; AC, arterial compliance; β, beta stiffness index; Ep, elastic modulus; PWVβ, carotid pulse wave velocity; cSBP, central systolic blood pressure; aPWV, aortic pulse wave velocity; MET, metabolic equivalent. Significant results are marked in bold*.

* *Adjusted for age, sex, body surface area, training years, and bSBP*.

+ *Adjusted for age, sex, body surface area, and training years*.

**Figure 1:**
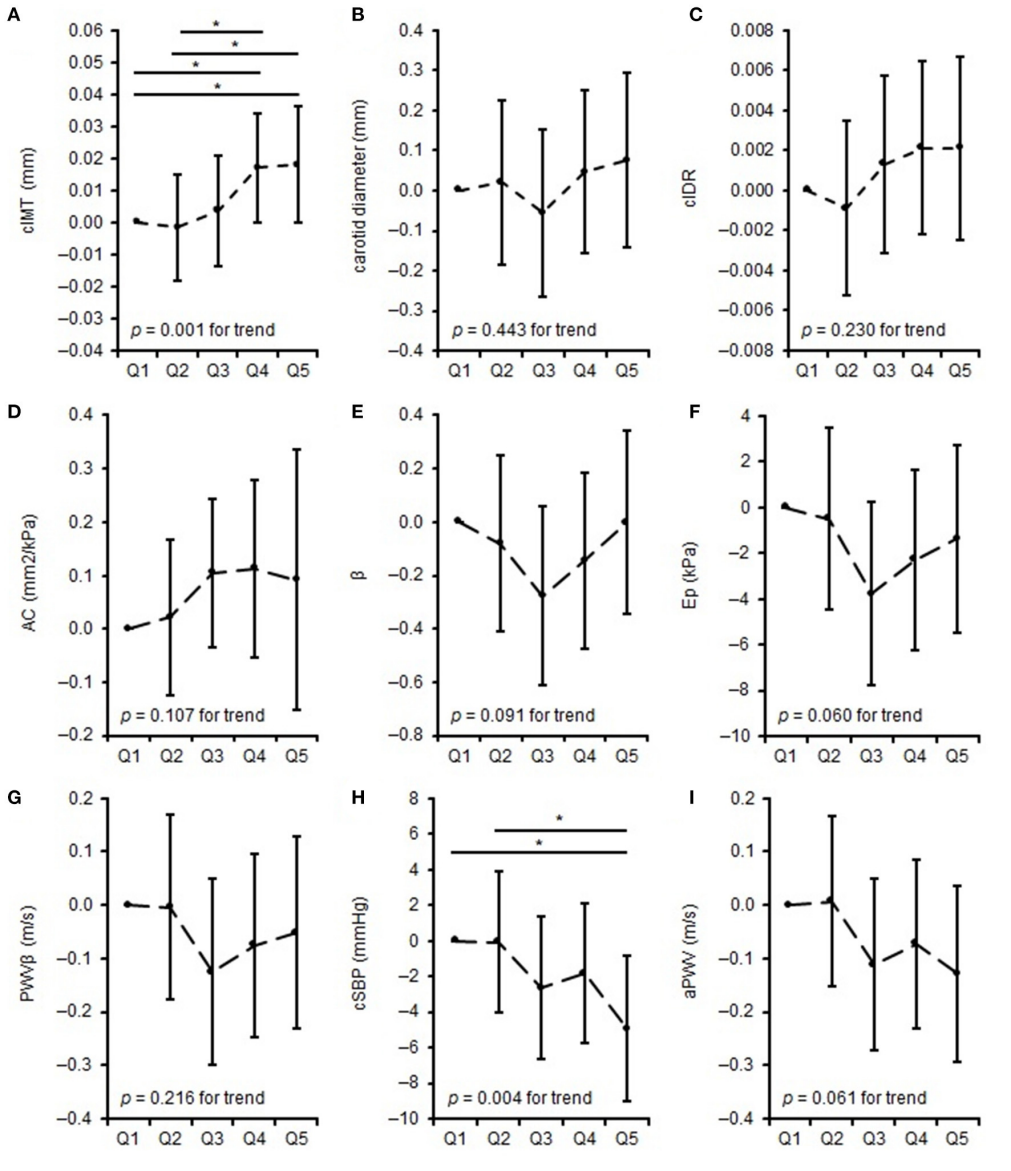
Differences in measures of vascular properties: mean difference to the lowest quintile of training duration. **(A–C)** represent differences in carotid arterial structure, **(D)** represents differences in carotid arterial elasticity, **(E–G)** represents differences in carotid arterial stiffness, **(H,I)** represents differences in central vascular function across quintiles of training duration. *when *p* < 0.05. cIMT, carotid intima-media thickness; cIDR, carotid intima-media thickness:carotid diameter-ratio; AC, arterial compliance; β, beta stiffness index; Ep, elastic modulus; PWVβ, carotid pulse wave velocity; cSBP, central systolic blood pressure; aPWV, aortic pulse wave velocity.

The differences in parameters of carotid arterial structure (adjusted for age, sex, BSA, and bSBP) and arterial function (adjusted for age, sex, and BSA) between quintiles of training duration are shown in Figure 1. Q5 and Q4 displayed thicker cIMT than Q1 (Q5 vs. Q1: *p* = 0.035, Q4 vs. Q1: *p* = 0.034) and Q2 (Q5 vs. Q2: *p* = 0.012, Q4 vs. Q2: *p* = 0.012). Furthermore, Q5 showed lower cSBP compared to Q1 (*p* = 0.008) and Q2 (*p* = 0.007). The ANCOVA model showed a tendency of significance for β, Ep and aPWV. There were no differences between the quintiles regarding carotid diameter, cIDR, AC, and PWVβ. Separate quintile plots for girls and boys are provided in the Supplementary Material.

**Figure 2:**
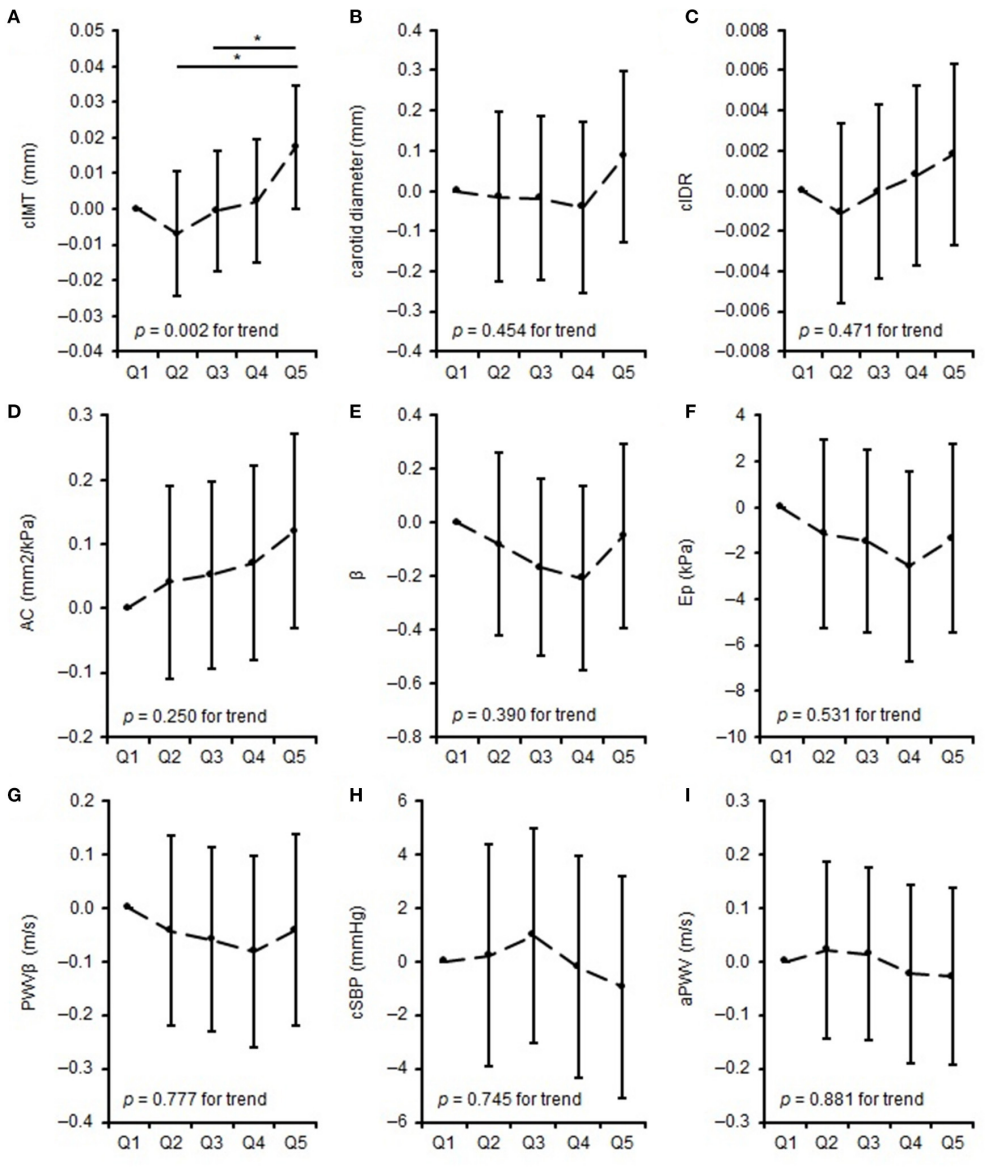
Differences in measures of vascular properties: mean difference to the lowest quintile of training intensity. **(A–C)** represent differences in carotid arterial structure, **(D)** represents differences in carotid arterial elasticity, **(E–G)** represents differences in carotid arterial stiffness, **(H,I)** represents differences in central vascular function across quintiles of training intensity. *when *p* < 0.05. cIMT, carotid intima-media thickness; cIDR, carotid intima-media thickness:carotid diameter-ratio; AC, arterial compliance; β, beta stiffness index; Ep, elastic modulus; PWVβ, carotid pulse wave velocity; cSBP, central systolic blood pressure; aPWV, aortic pulse wave velocity.

The differences in parameters of carotid arterial structure (adjusted for age, sex, BSA, and bSBP) and arterial function (adjusted for age, sex, and BSA) and quintiles of training intensity are shown in Figure 2. Q5 displayed thicker cIMT than Q2 (*p* = 0.001) and Q3 (*p* = 0.029). There were no differences between the quintiles in carotid diameter, cIDR, AC, β, Ep, PWVβ, cSBP, and aPWV. Separate quintile plots for girls and boys are provided in the Supplementary Material.

## Discussion

We found divergent results regarding the association between training duration and intensity and vascular properties in active children and adolescents. Overall, higher training duration and intensity correlated with higher IMT and elasticity of the carotid artery. Higher training duration was related to better central hemodynamics and higher training intensity to larger carotid diameter. Sex-specific analyses revealed better carotid arterial elasticity with higher training duration in boys and girls. Only boys showed better carotid arterial elasticity with higher training intensity and higher training duration with better central hemodynamics. Additionally, higher training duration and intensity was associated with thicker cIMT in boys. Girls, however, showed lower carotid arterial stiffness with higher training duration.

Besides the linear associations, the quintile analyses indicated that active children and adolescents with very high training duration and intensity have a significantly thicker cIMT than children and adolescents with lower training duration and intensity. Furthermore, active children and adolescents with very high training duration showed significantly lower cSBP.

### Carotid Artery Structure

cIMT measurement in children and adolescents is mainly used for cardiovascular risk stratification (35). Compared with findings by Weberruß et al. (22) (0.46 ± 0.03 mm in boys and girls), mean cIMT of girls (0.46 ± 0.04 mm) is at a similar level, but mean cIMT of boys (0.48 ± 0.04 mm) is slightly higher in our cohort.

To our knowledge, only Demirel et al. (12) investigated cIMT in an active pediatric population and found lower cIMT in adolescent male wrestlers than age- and sex-matched controls. The comparison of cIMT values indicated a substantial deviation from cIMT observed in this study to cIMT values of Demirel et al. (12) (0.28 mm in athletes, 0.35 mm in controls). This distinction is probably caused by the different measurement methodology, which is a major determinant in cIMT assessment: cIMT measured directly after the bulb is larger than 2–3 cm proximal the bifurcation (36). Demirel et al. (12) measured only on the right carotid artery and 2 cm proximal to the bifurcation, which might explain the deviation in cIMT. cIMT sonography guidelines were published in 2015 to enable the comparison of different studies. These guidelines recommend measuring the cIMT 1 cm proximal to the carotid bulb (35).

Further studies in adult athletes observed lower (37), higher (38), or similar cIMT (39) compared with controls. In general, physical activity has a beneficial effect on the cardiovascular system of individuals. However, the positive effect seems to be reversed with an excessively high activity volume, as several studies have reported adverse effects (4, 10). The U-shaped or reverse J-shaped curve describes that individuals benefit from exercise, even if the exercise exceeds the level recommended for health maintenance and health promotion, until a turning point is reached and positive effects of exercise are reversed at very high exercise loads. In other words, the health benefits of exercise in individuals with an extremely high exercise load can be reversed to the approximate levels of inactive individuals, thus, high and intensive exercise can be of disadvantage (9).

Arem et al. (40) described a dose-response relationship of exercise levels per week and mortality risk, analogous to Eijsvogels et al. (9), which depicted a reduction in the mortality risk with higher exercise volume but a revision of the trend and increase of mortality risk at an exercise volume of ≥ 75 MET h/week. In our study, higher training duration and intensity was both related to a thicker cIMT, independent of the number of years the participants have been exercising. Furthermore, concerning training duration, both Q4 and Q5 showed significantly higher cIMT compared to Q1 and Q2. This means that children and adolescents who trained at least 8 h per week in a sports club had thicker cIMT. Concerning training intensity, a gradual increase from Q2 (> 48.45 MET-hours-index) was observed and children and adolescents in Q5 (>99 MET h/week) showed a significantly greater wall thickness compared to Q2 (Δ0.018 mm) and Q3 (Δ0.024 mm) compared to their peers.

cIMT is only a surrogate for subclinical vascular changes but independently associated with a higher risk for cardiovascular mortality (41). This emphasizes cIMT as an important parameter in assessing cardiovascular health, not only in patients but also in asymptomatic subjects – starting to screen them at a young age to prevent cardiovascular events in the future. Regarding our cohort, all participants are children and adolescents who are actively training in sports club activities. Therefore, our study participants are different from other studies investigating vascular changes in patients or inactive subjects and, therefore, cannot be compared to these studies. Although the study design does not allow to assume a dose-response relationship between training load and cIMT in our study collective, the results show that intensive exercise is associated with a thicker vessel wall.

During intensive exercise, the carotid artery diameter increases steadily by 6%, while after exercise the diameter decreases by 3% below the baseline level. One hour after exercise, the diameter is again 3% above the baseline level (42). Therefore, our result that higher carotid diameter was observed with higher training intensity might suggest that this increase in carotid diameter persists after exercise. Thijssen et al. (43) observed a similar diameter of the carotid artery in young adults after an 8-week cycling training. Rowley et al. (44) compared three arteries between upper limb athletes, lower limb athletes, and controls. They found that the effect of exercise on the vessel diameter was locally situated at the exercising limb. They observed larger brachial artery diameter in upper limb athletes, larger superficial femoral artery diameter in lower limb athletes, and similar carotid artery diameter in upper and lower limb athletes compared with controls. To what extent the results of Rowley et al. (44) can already be observed in childhood and adolescence should be clarified.

Also, we observed no correlation between the cIDR and training frequency and training intensity in our active children and adolescents. In contrast to our study results, Rowley et al. (44) reported significantly lower wall-to-lumen ratio of carotid, superficial femoral, and brachial arteries in upper and lower limb athletes than controls. Furthermore, Thijssen et al. (43) observed a significant reduction of the wall-to-lumen ratio of the carotid and superficial femoral artery after an 8-week cycling training. In our study, we did not differentiate between local physiological stimuli of different types of sports, e. g. measurement of vascular properties at the superficial femoral artery in soccer players or measurement of vascular properties at the brachial artery in rowers. It is conceivable that the chronic exertion of endurance or strength sports or sports that involved the upper or lower body may lead to a different form of adaptation of the vascular system to exercise at a young age. Furthermore, the type of sport's influence on the adaptation of structural properties is also of interest in this context. However, it was not possible in our study to investigate differences in vascular properties between types of sport because children and adolescents from many different types of sports participated in the study.

The adaptation of vascular properties as a result of exercise is related to the increase in blood flow, the associated increase in shear stress and influence of vasodilators (NO, eNOS), synthesized by endothelial cells (45, 46). Therefore, in our study, we cannot determine to what extent other factors, such as metabolic or vasoactive substances, change vascular properties at a young age as a result of exercise.

### Carotid and Central Vascular Function

Children and adolescents in our study have a higher carotid arterial elasticity (AC, 1.55 mm^2^/kPa in boys and 1.45 mm^2^/kPa in girls) and lower carotid arterial stiffness (Ep, 37.4 kPa in boys and 38.2 kPa in girls; β, 3.18 in boys and 3.28 in girls; PWVβ, 3.60 m/s in boys and 3.65 m/s girls) than the cohort of Weberruß et al. (22), which consisted of healthy children assessed in a school setting (AC, 1.2 mm^2^/kPa in boys and girls; Ep, 46.6 kPa in boys and 44.9 kPa in girls; β, 3.9 in boys and 3.8 in girls; PWVβ, 4.0 m/s in boys and girls). Even though our study collective is ~2 years older, these comparisons support the evidence that physical activity is favorably associated with carotid arterial elasticity in children and adolescents (14). In contrast, our study participants showed a higher cSBP (105.9 mmHg in boys, 102.5 mmHg in girls) and a higher aPWV (4.93 m/s in boys, 4.74 m/s in girls) than Elmenhorst et al. (34) (cSBP, 100.76 mmHg boys and 100.5 mmHg in girls; aPWV, 4.7 m/s in boys and 4.6 m/s in girls). Differences in elasticity between proximal and distal arteries might be explained by the fact that vascular smooth muscle cells along the arterial tree are derived from different sources of progenitor cells (47). For example, the vascular smooth muscle cells in the CCA develop from the neural crest, while the vascular smooth muscle cells in the dorsal aorta have their embryonic origin from somites (47, 48). These differences do not only occur between different vessels but even between individual segments of a single vessel (47). However, when comparing aPWV and PWVβ, it should be considered that aPWV was measured oscillometrically at the brachial artery and calculated by the three-level algorithm, and PWVβ was measured by ultrasound at the carotid artery. Therefore, it is important to consider the localization of the measurement (central or carotid) when interpreting arterial stiffness.

In our study collective, we observed better carotid arterial elasticity correlated with both higher training duration and intensity. Overall, we could not find an association between parameters of carotid arterial function and training duration or intensity. However, the results of the quintile analyses of training duration suggest that β, Ep, and PWVβ follow an approximately U-shaped pattern. The parameters decreased between Q1 and Q3, which indicated a reduction in carotid arterial stiffness, whereas from Q3 to Q5, parameters increased again, which implied higher carotid arterial stiffness. In Q5, the carotid arterial stiffness parameters were below (Ep and PWVβ) or at a comparable level (β) to Q1, which led to the conclusion that carotid arterial stiffness of children and adolescents in Q5 was improved (Ep and PWVβ) or comparable (β) with that of children and adolescents in Q1.

A comparison of previous studies on the association between arterial stiffness and physical activity or exercise in children and adolescents revealed a heterogeneous pattern. Ried-Larsen et al. (49) observed lower values of carotid Young's elastic modulus and stiffness index with higher moderate-to-vigorous physical activity. Weberruß et al. (22) assessed carotid arterial stiffness using the same protocol and equipment in *n* = 870 children and adolescents. In a sub-sample of *n* = 697 subjects (376 girls), cardiorespiratory fitness was significantly associated with carotid arterial stiffness parameters. No significant associations were obtained for muscular strength. In a one-way variance analysis, very fit boys and girls (>80th percentile for cardiorespiratory fitness) had significantly lower carotid arterial stiffness parameters compared with low fit subjects (<20th percentile). In contrast, other studies could not find a link between carotid arterial stiffness and physical activity in children and adolescents (14). Also, Demirel et al. (12), who compared adolescent wrestlers with controls, did not find significant differences in carotid arterial stiffness parameters. In contrast, in physically active adults, higher carotid arterial stiffness was observed in swimmers than cyclists, lower arterial stiffness in cyclists than controls, and similar carotid arterial stiffness between swimmers and controls (50). Therefore, carotid arterial stiffness also seems to be influenced by the type of sport performed.

We observed a lower cSBP and aPWV and thus a better central vascular function associated with a higher training duration but not with training intensity. Furthermore, the lower bSBP in Q5 of training duration compared to Q1 und Q2 confirms the findings of the linear regression analyses and depicts improved central arterial function in children and adolescents of very high training load. There was a tendency but not a significant difference between quintiles of training duration and aPWV, with the curve of aPWV being very similar to the curve of bSBP.

Previous studies on the relationship between physical activity in everyday life and central arterial stiffness have found little to no correlation in children and adolescents (14). However, the fact that targeted physical activity can affect central arterial stiffness is indicated by a comparison of extremely frequent physical activity with controls. Although Bauer et al. (51) observed lower cSBP in elite handball players, Vlachopoulos et al. (5) found higher cSBP and aPWV in marathon runners, both studies compared to controls.

However, it seems that the type of exercise plays a decisive role in this context. D'Andrea et al. (6) observed 190 strength athletes, 220 endurance athletes, and 240 controls. They found better aortic distensibility in endurance athletes compared to strength athletes and controls but higher aortic stiffness in strength athletes than endurance athletes and controls. Furthermore, they concluded that training duration and endurance training have a significant positive impact on aortic distensibility. In contrast, training duration and strength training have a significant negative impact on aortic stiffness. Also, a targeted 12-week strength training in powerlifting athletes increased cSBP (52). In addition to the type of training and the training duration, the training intensity also influences vascular function. Vlachopoulos et al. (5) found that the intensity of exercise in marathon running is related to a significantly higher aPWV, corrected for mean pressure.

### Differences Between Boys and Girls

We observed sex-specific differences in vascular properties, although boys and girls were of similar age. Boys showed thicker cIMT, larger carotid diameter and cIDR, higher central hemodynamics and carotid arterial elasticity compared to girls, which is in contrast to a German reference cohort (22). While the positive relationship between AC and training duration was confirmed in both girls and boys according to the overall analysis, better carotid arterial elasticity in correlation with training intensity was only observed in boys. Controversially, only girls showed lower carotid arterial stiffness (β and Ep) associated with higher training duration, while only boys showed improved central hemodynamics associated with higher training duration. The positive association between cIMT and training duration and intensity was also confirmed only in boys.

Sex differences are, in general, difficult to interpret according to chronological age, as vascular properties aligned with the maturation stage (53). In boys, puberty generally starts later than in girls and in young athletes later than in their peers (54, 55). Even if an exact determination of the maturity status was not obtained, it can be assumed that a large majority of the included subjects are currently in the puberty phase due to the medium age of 14.0 ± 1.94 years. In our study setting, it was not possible to determine the puberty status based on the Tanner stages, because the study participants visited our outpatient clinic for a fee-based sports medical examination and not for study purposes only.

Differences in hormone levels between boys and girls might explain the differences in vascular properties. Testosterone is released in boys during puberty and increases the activation of the sympathetic nervous system and renin-angiotensin system, which is associated with increased SBP (56, 57). Furthermore, higher testosterone levels are associated with higher arterial stiffness and left ventricular mass, which in itself is associated with higher cIMT (58–60). In contrast, estradiol and progesterone levels, which are higher in girls compared to boys, have a cardioprotective effect (61–64).

### Future Directions

It is necessary to clarify whether the relationship between cIMT and higher training load manifests itself with the continuation of training activities or whether the adolescent body adapts to the training loads and the vascular properties normalize as a result. In addition, the relationship between vascular and cardiac properties in young active subjects should be further clarified to preclude possible adverse effects of thickened cIMT with higher training load on cardiac structure and function. Furthermore, measuring cardiovascular endpoints is difficult in this age group. Therefore, cohort studies should investigate whether alterations in vascular properties that already occur in youth as a result of exercise lead to a higher risk of cardiovascular disease and mortality in the future. Moreover, vascular properties should be compared between different sports to investigate the effects of sport-dependent training loads. In this context, not only the vascular properties of the CCA should be investigated, but also the properties of the femoral and brachial artery, since different sports affect different areas of the body. Future studies should also investigate whether the sex differences in the relationship between vascular properties and exercise duration and intensity have a hormonal explanation.

### Limitations

Some limitations affect our study results. Owing to the cross-sectional design, it is not possible to make a causal statement regarding the cause-and-effect relationship between the duration and intensity of exercise and vascular properties. Therefore, we will investigate this aspect in a further longitudinal study, paying attention to whether a permanently high level of activity leads to changes in vascular properties (15). Our study collective consisted of children and young people who were regularly active in sports clubs and did not include a control group with inactive children and adolescents. Therefore, it is not possible to assess whether active and inactive children and adolescents differ in vascular properties. We assessed weekly training duration and intensity using a questionnaire. Compared with accelerometers, questionnaires measure subjective and not objective activities. Therefore, the validity of the data obtained by questionnaires depends on the respondents' ability to remember and the accuracy of the information provided. In order to collect the exercise behavior as exactly as possible, the questionnaire was explained in detail and help was offered. A further limitation is the missing assessment of the maturity status because it is known that maturity status influences the vascular properties of children and adolescents (53).

Furthermore, we did not measure biochemical parameters, such as nitric oxide, that have been reported to influence the relationship between exercise and vascular properties (65). The regression models of carotid arterial function revealed a poor model fit. In contrast, the regression models of the cIMT with about 20% and the regression models of central arterial function with more than 40% (total and boys) revealed an acceptable model fit. Independently of our association analyses, other factors, which we have not recorded, exist which forecast the carotid arterial function of active children and adolescents. Therefore, future studies should include parameters such as maturity status and biochemical markers.

## Conclusion

We studied for the first time the association between training duration and intensity and vascular properties and sex-specific differences in these associations in a study collective of active children and adolescents. The novelty findings of our study are a negative association between training load and cIMT, but better carotid arterial elasticity in physically active children and adolescents. Furthermore, central hemodynamics were improved with higher training duration, whereas girls showed lower carotid arterial stiffness. Therefore, we assume a muscular and also functional adaptation as a result of intensive exercise. In future, children and adolescents with extremely high training loads should be regularly examined with regard to their vascular properties in order to detect possible negative and persistent effects of competitive sports on vascular health at an early stage.

## Data Availability Statement

The datasets analyzed within the study are available from the corresponding author on reasonable request.

## Ethics Statement

The studies involving human participants were reviewed and approved by Technical University of Munich. Written informed consent to participate in this study was provided by the participants' legal guardian/next of kin.

## Author Contributions

LB, HW, TS, and RO-F designed the study. LB and TE performed the data collection. LB performed the data analysis and drafted the manuscript. All authors contributed to the article and approved the submitted version.

## Conflict of Interest

The authors declare that the research was conducted in the absence of any commercial or financial relationships that could be construed as a potential conflict of interest.
