# Supplementary material for: Exercise Training Duration and Intensity Are Associated With Thicker Carotid Intima-Media Thickness but Improved Arterial Elasticity in Active Children and Adolescents
Source: Front Cardiovasc Med. 2021 Jul 8;8:618294. doi: 10.3389/fcvm.2021.618294 (PMC8295565; doi:10.3389/fcvm.2021.618294)
Supplement: Supplementary file 1 [file Data_Sheet_1.docx]

Supplementary Material


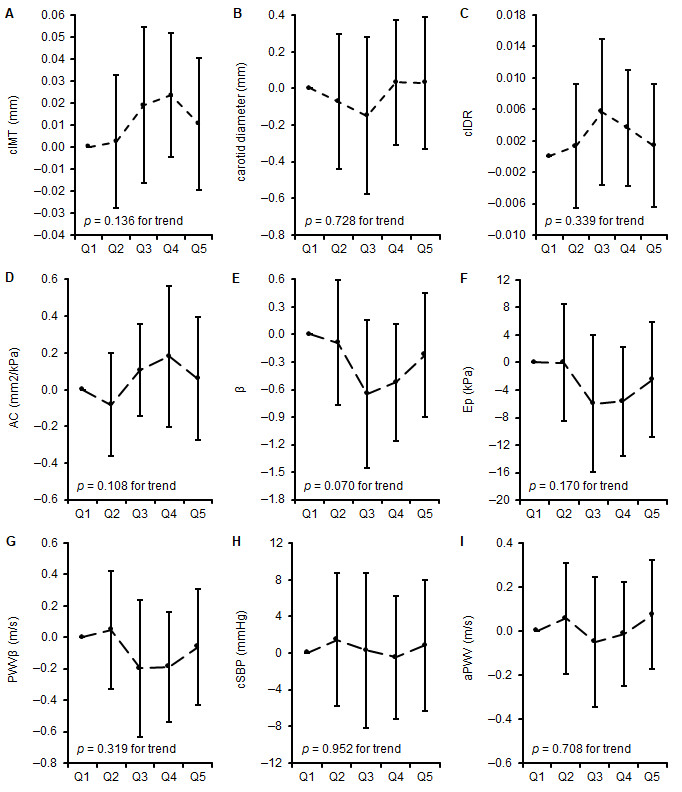


**Supplementary Figure 1.** Differences in measures of vascular properties: mean difference to the lowest quintile of training duration in girls. **A - C** represent differences in carotid arterial structure, **D** represents differences in carotid arterial elasticity, **E-G** represents differences in carotid arterial stiffness, **H-I** represents differences in central vascular function across quintiles of training duration. * when *p* < 0.05. cIMT, carotid intima-media thickness; cIDR, carotid intima-media thickness:carotid diameter-ratio; AC, arterial compliance; β, beta stiffness index; Ep, elastic modulus, PWVβ, carotid pulse wave velocity; cSBP, central systolic blood pressure; aPWV, aortic pulse wave velocity.


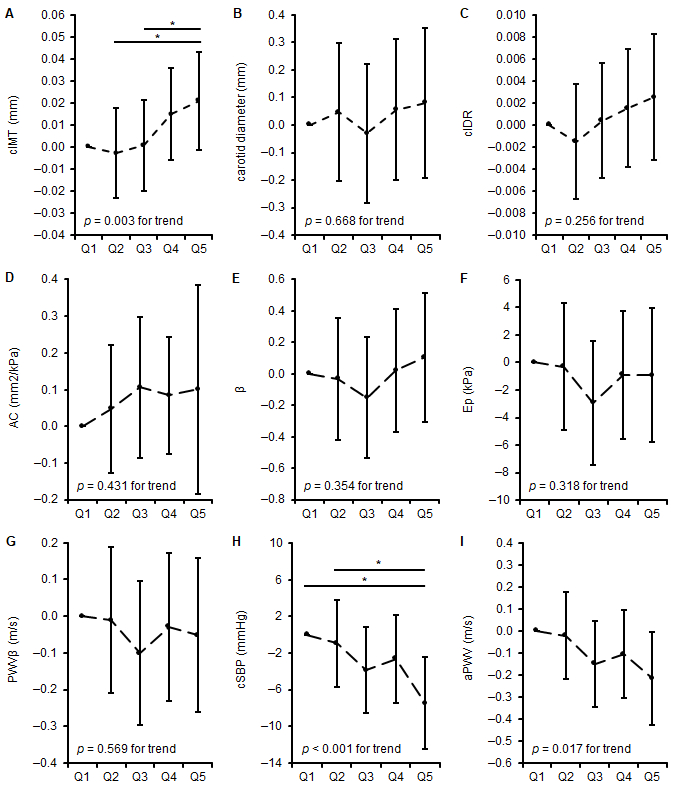


**Supplementary Figure 2.** Differences in measures of vascular properties: mean difference to the lowest quintile of training duration in boys. **A - C** represent differences in carotid arterial structure, **D** represents differences in carotid arterial elasticity, **E-G** represents differences in carotid arterial stiffness, **H-I** represents differences in central vascular function across quintiles of training duration. * when *p* < 0.05. cIMT, carotid intima-media thickness; cIDR, carotid intima-media thickness:carotid diameter-ratio; AC, arterial compliance; β, beta stiffness index; Ep, elastic modulus, PWVβ, carotid pulse wave velocity; cSBP, central systolic blood pressure; aPWV, aortic pulse wave velocity.


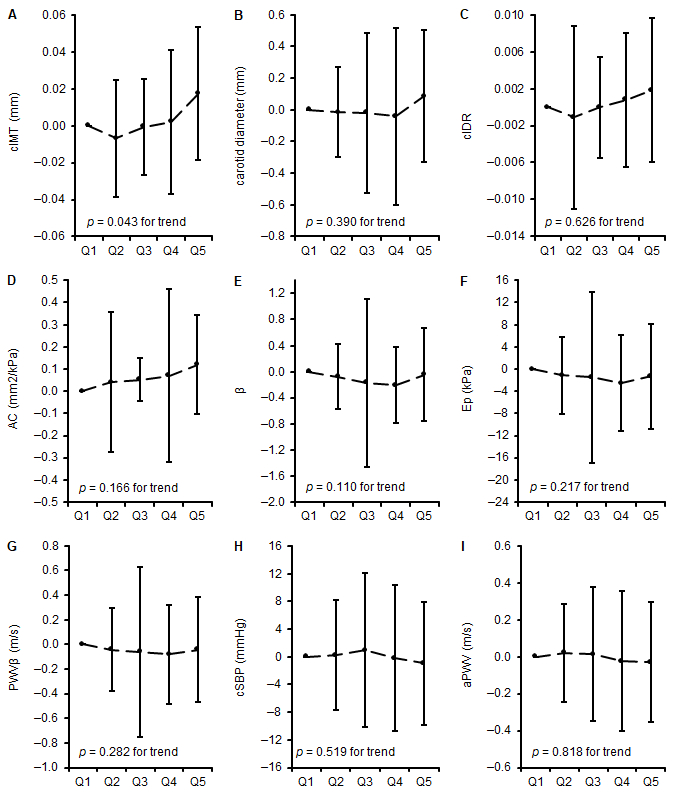


**Supplementary Figure 3.** Differences in measures of vascular properties: mean difference to the lowest quintile of training intensity in girls. **A - C** represent differences in carotid arterial structure, **D** represents differences in carotid arterial elasticity, **E-G** represents differences in carotid arterial stiffness, **H-I** represents differences in central vascular function across quintiles of training duration. * when *p* < 0.05. cIMT, carotid intima-media thickness; cIDR, carotid intima-media thickness:carotid diameter-ratio; AC, arterial compliance; β, beta stiffness index; Ep, elastic modulus, PWVβ, carotid pulse wave velocity; cSBP, central systolic blood pressure; aPWV, aortic pulse wave velocity.


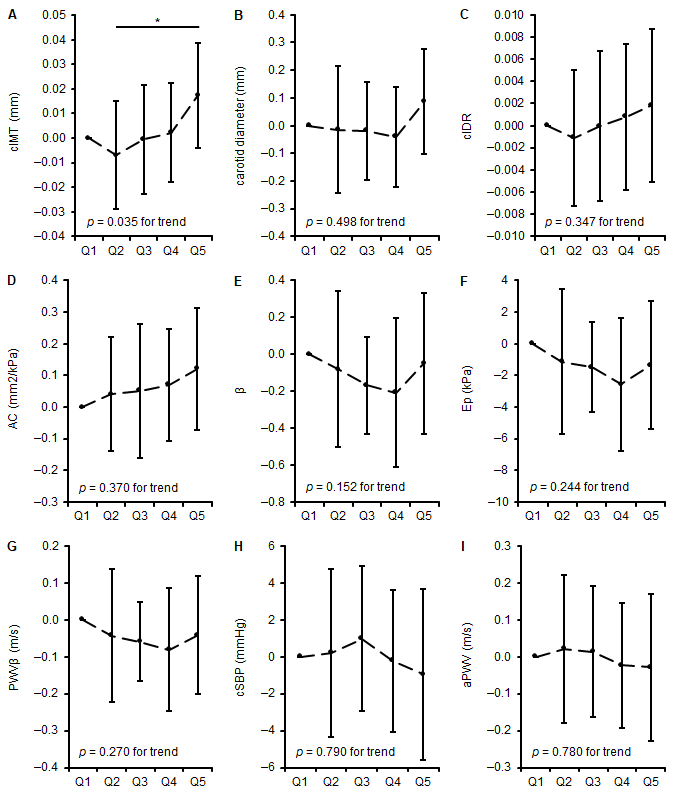


**Supplementary Figure 4.** Differences in measures of vascular properties: mean difference to the lowest quintile of training intensity in boys. **A - C** represent differences in carotid arterial structure, **D** represents differences in carotid arterial elasticity, **E-G** represents differences in carotid arterial stiffness, **H-I** represents differences in central vascular function across quintiles of training duration. * when *p* < 0.05. cIMT, carotid intima-media thickness; cIDR, carotid intima-media thickness:carotid diameter-ratio; AC, arterial compliance; β, beta stiffness index; Ep, elastic modulus, PWVβ, carotid pulse wave velocity; cSBP, central systolic blood pressure; aPWV, aortic pulse wave velocity.
